# 

**Published:** 1893-05

**Authors:** 


CONTENTS.

Vol. XL No. 5.

| | PAGE. |
|---|---|
| What is Cholera | 97 |
| Poisonous Milk | 102 |
| Coolings | 104 |
| The Living and the Dead | 106 |
| Spring Diseases | 109 |
| Proper Care of Infants | 110 |
| A Surfeit | 112 |
| Warts | 113 |
| Onions | 113 |
| The Difference | 113 |
| Sneezes and Stitches | 113 |
| Behind the Counter | 114 |
| Cases of Hypnotic Suggestion | 116 |
| Medical "Etiquette" | 117 |
| Editor's Outlook | 118 |
| Literary | 119 |
| The Garden at My Door | VIII |

INDEX TO ADVERTISEMENTS OF HALF A PAGE AND OVER.

| | PAGE. |
|---|---|
| Dr. Jaeger's Sanitary Woolens | I |
| National Mutual Insurance Co. | II |
| The Press Claims Co. | III |
| Chambers' Encyclopedia | IV |
| Recreation Bureau | VII |
| Sterlingworth Sanitarium | VIII |
| J. Fehr's Compound Talcum. | IX |
| Herba Vita. | X |
| Royal Baking Powder | XI |
| Buffalo Lithia Water | XI |

